# Supplementary material for: River channel connectivity shifts metabolite composition and dissolved organic matter chemistry
Source: Nat Commun. 2019 Jan 28;10:459. doi: 10.1038/s41467-019-08406-8 (PMC6349891; doi:10.1038/s41467-019-08406-8)
Supplement: Supplementary file 1 — Supplementary Information [file 41467_2019_8406_MOESM1_ESM.pdf]

Supplementary Information

River channel connectivity shifts metabolite composition and dissolved organic matter chemistry

Lynch et al.

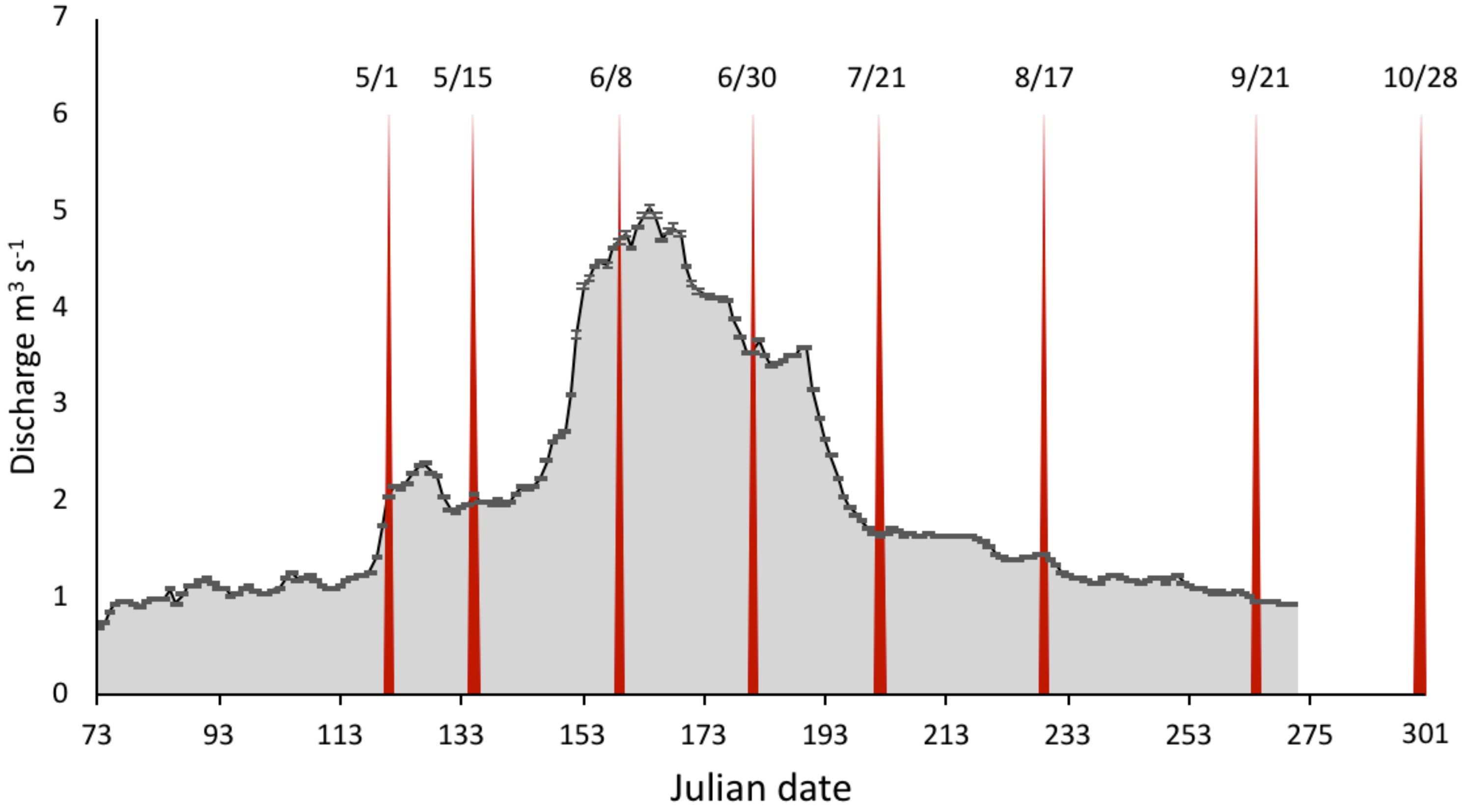

Supplementary Figure 1 | Volumetric daily mean discharge at the beaver meadow outflow, located within North St. Vrain watershed. Vertical, red lines indicate the eight sampling periods. The sharp rise in discharge in May is driven by seasonal snowmelt. Capacitance rods were removed after September 28 (271 Julian days); hydrological data are therefore not available for the final sampling period (October 28, 2015), but flows continued to decline throughout the fall.

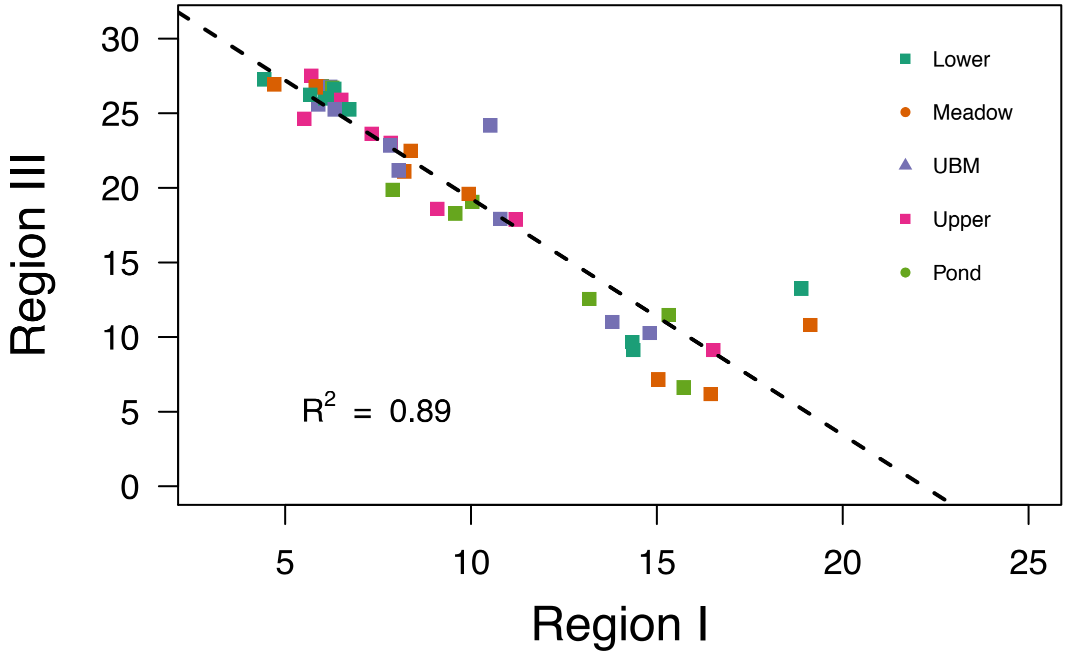

Supplementary Figure 2 | Linear regression of EEMS region III (fulvic-type acids) against EEMS region I (soluble microbial-type proteins).

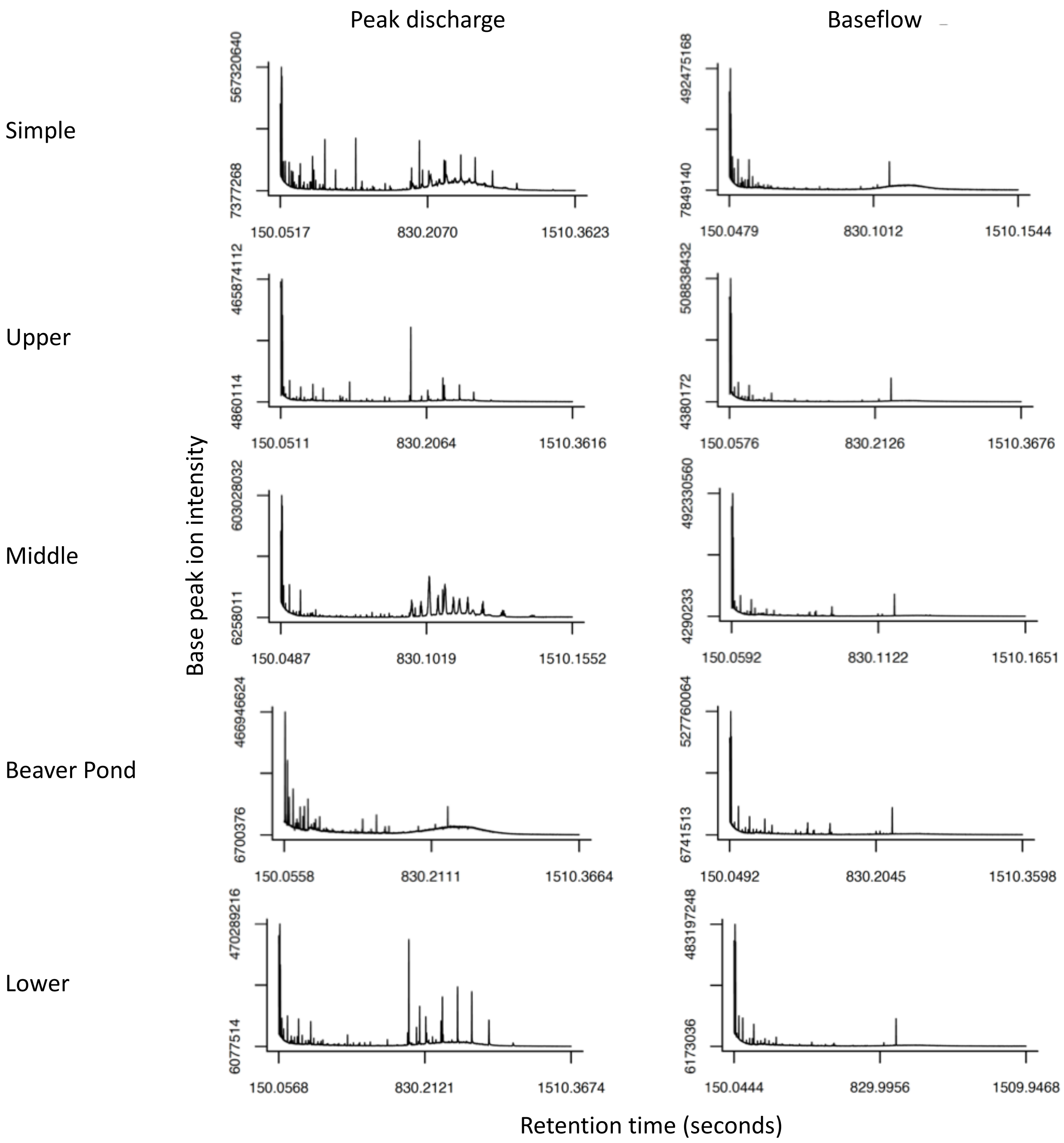

Supplementary Figure 3 | Total ion chromatographs of representative samples across each landscape position collected during peak discharge (left column) or baseflow (right column). The concentration of metabolites exported across the hydrologically connected landscape is high during snowmelt (May 15, 2015), but the variability in metabolite composition diverges across landscape position as flows subside.

a)

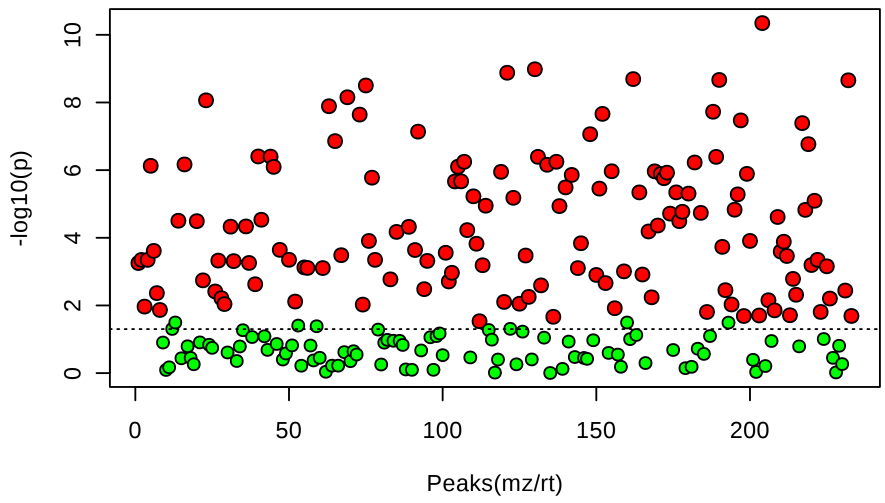

b)

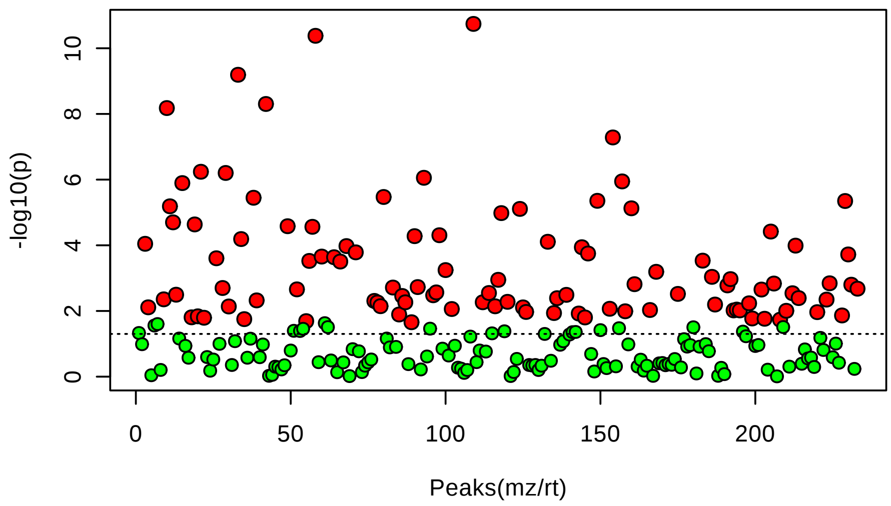

Supplementary Figure 4 | Metabolites identified by ANOVA analysis that differ significantly across landscape position (panel a) and sampling period (panel b) are shown in red. Metabolites that are not significantly different across comparisons are shown in green (below the dashed line). Overall, 141 significant features were identified for landscape position and 106 significant features were identified for sampling period.

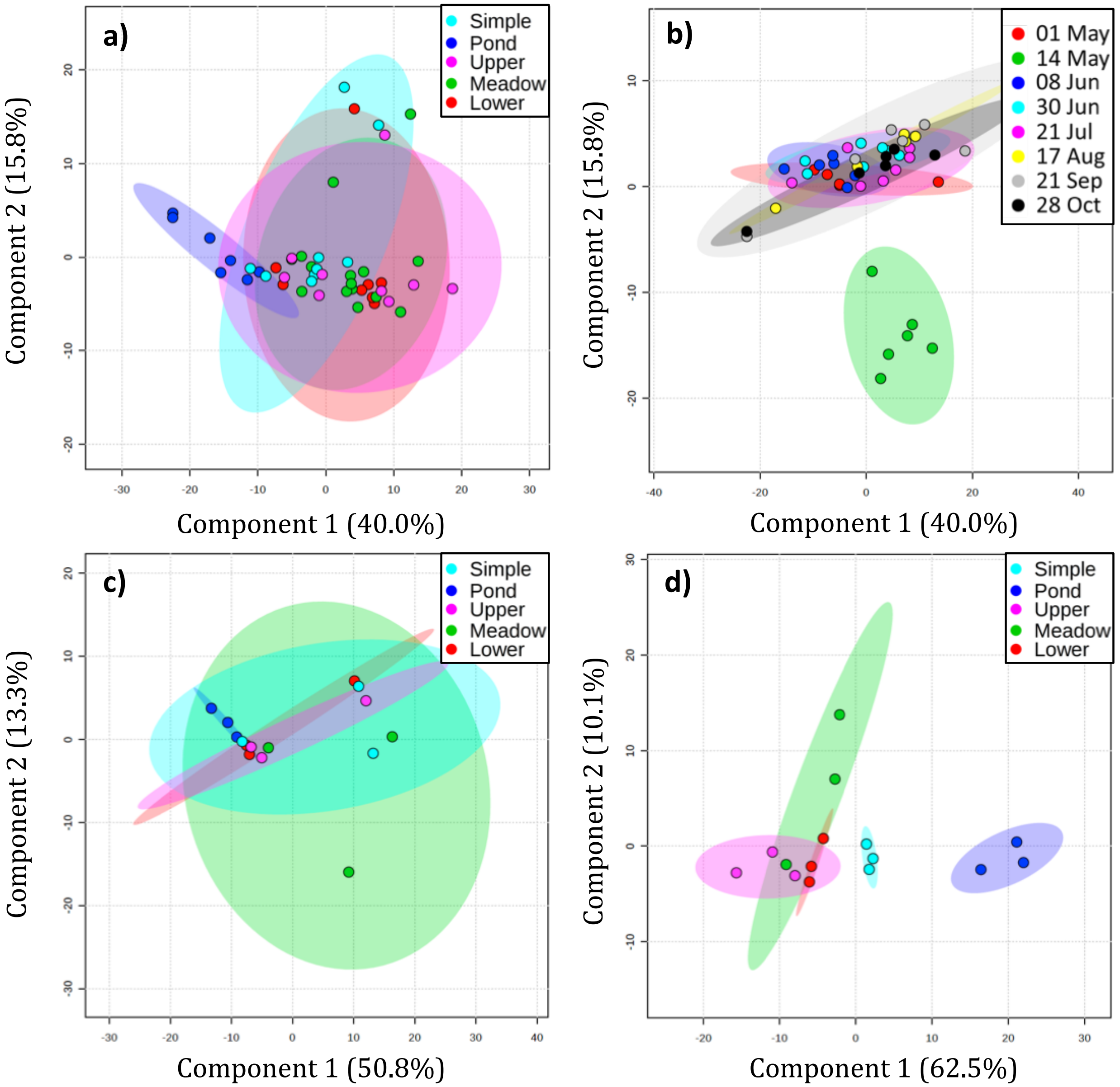

Supplementary Figure 5 | Unsupervised PCA plot of metabolites clustered by landscape position (a), sampling period (b), rising limb, including only May 01, May 15, June 08 (c), or base flow, including only Aug 17, Sep 21, Oct 28 (d). Each circle represents a sample and shaded ellipses represent 95% confidence intervals for each *a-priori* cluster. Ellipse overlap signifies no significant difference between clusters.

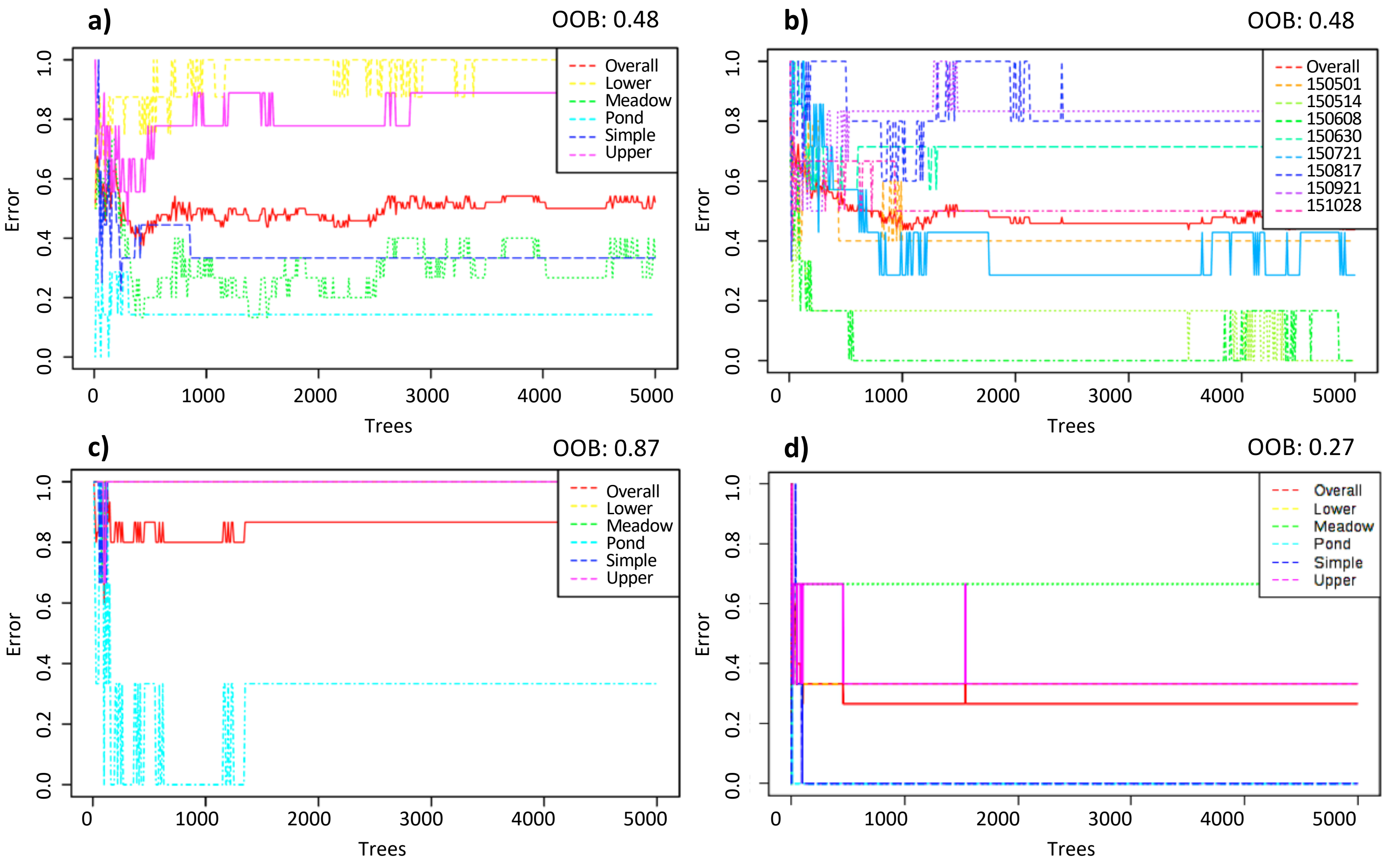

Supplementary Figure 6 | Classification results using random forest with 5,000 trees, where metabolites are clustered by landscape position (a), sampling period (b), rising limb, including only May 01, May 15, June 08 (c), or base flow, including only Aug 17, Sep 21, Oct 28 (d). The random forest approach eliminates the tendency of single decision tree models to overfit small datasets.[1,2] Each plot shows the shows the out-of-bag (OOB) error rates versus an increasing number of trees (overall OOB errors reported above each plot). OOB errors are greatest during peak flows, when organic matter is flushed through a hydrologically connected system, and lowest during low flow periods, when metabolite profiles diverge across the landscape.

a)

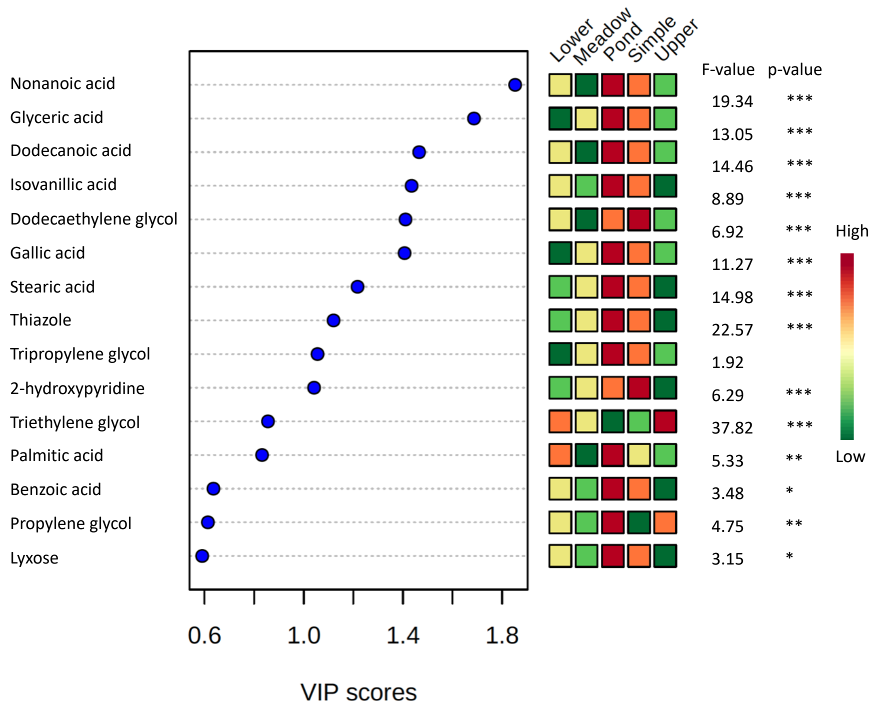

b)

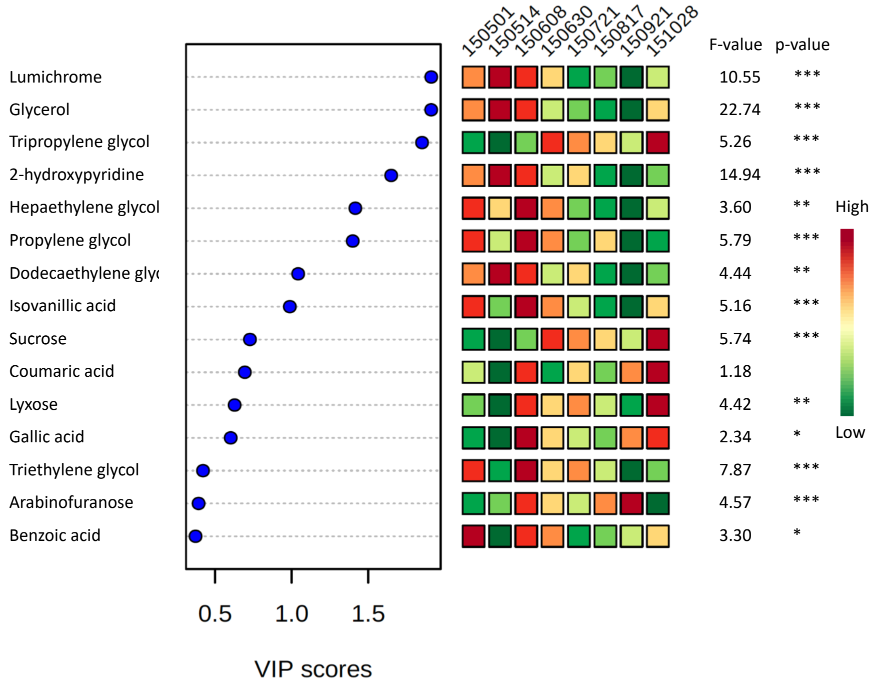

Supplementary Figure 7 | VIP (variable importance in projection) scores for the top 15 annotated metabolites separating groups by landscape position (panel a) and sampling period (panel b). F-statistics and p-values from multi-class ANOVA analysis are provided for reference. See Supplementary Table 3 for classification of annotated metabolites.

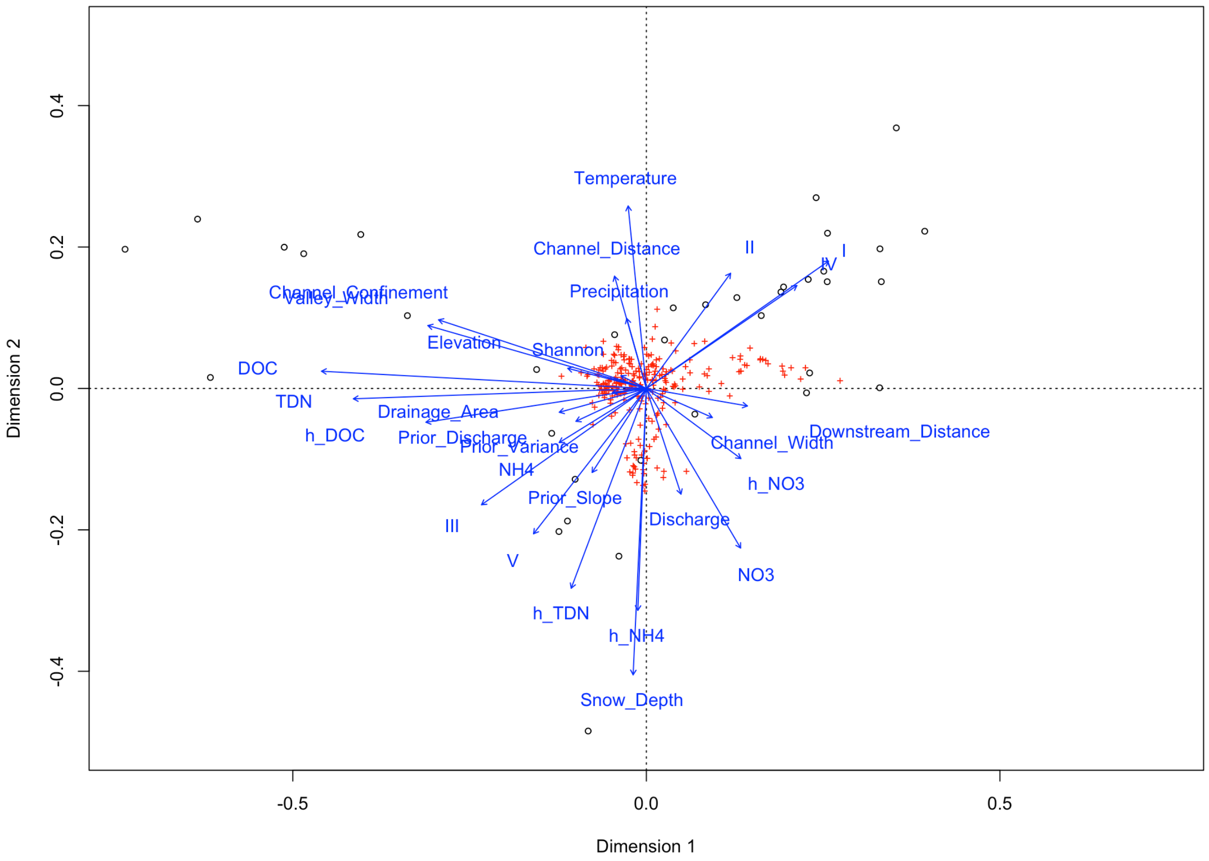

Supplementary Figure 8 | Multivariate analysis of metabolites and all potential environmental drivers using constrained analysis of principal coordinates. Ordinations are based on Bray-Curtis dissimilarity, computed from normalized peak intensities, and used to test for an association of metabolite composition (as derived from EI GC-MS) with spatial, climate, and fluvial characteristics. Red crosses represent individual metabolites, black circles represent samples, blue vectors represent potential predictor variables. DOC, dissolved organic carbon; TDN, total dissolved nitrogen; NH4, ammonium; NO3, nitrate; h preceding the prior variables indicate hyporheic rather than surface water samples; I-V, EEMS regions; Prior_Discharge, mean discharge or the three days prior to, and including the sampling date; Shannon, Shannon-Weiner diversity index.

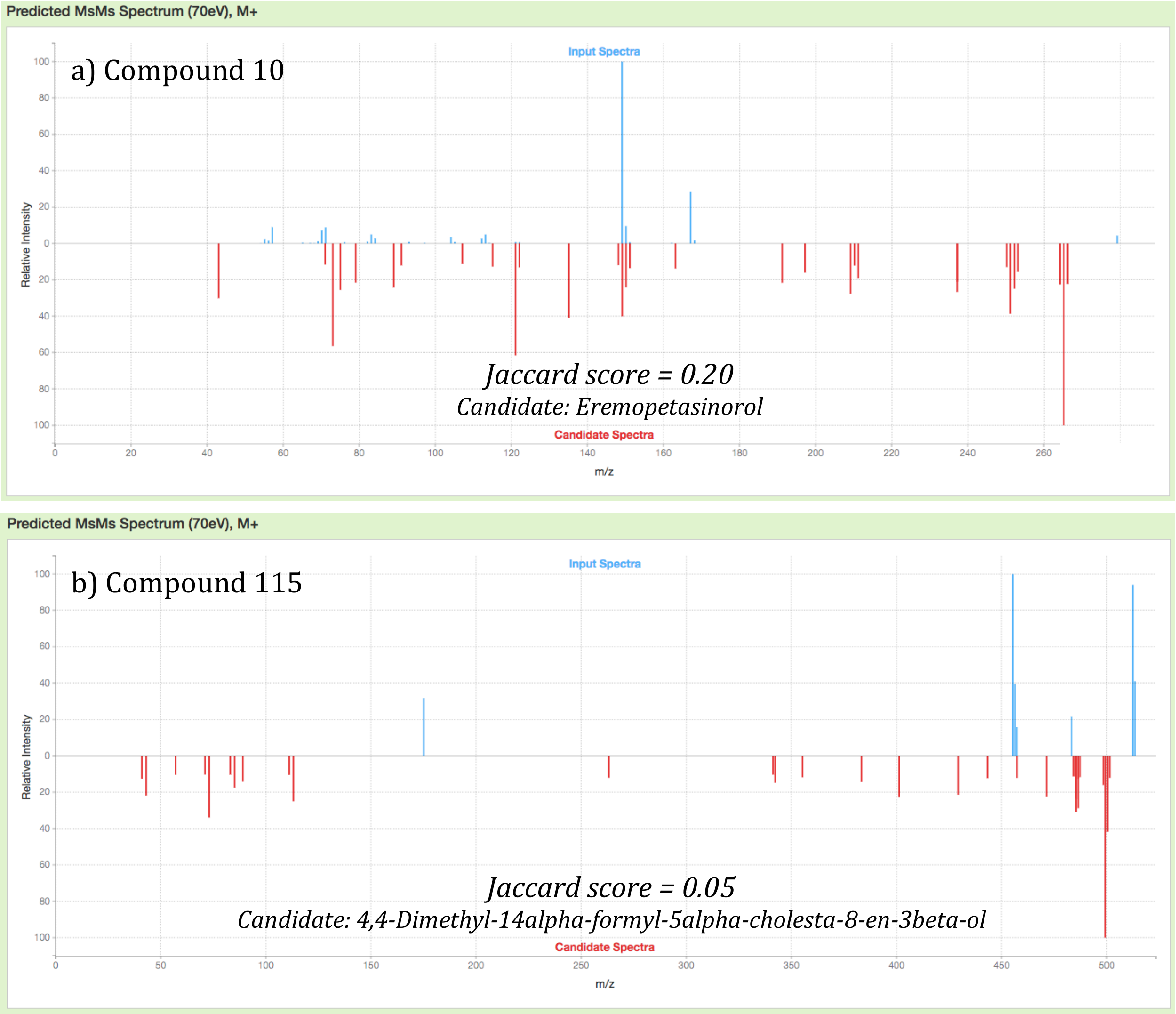

Supplementary Figure 9 | Spectral similarity between measured compounds (S_M, shown in blue) and the top-ranking predicted candidate spectra (S_P, shown in red) identified using Competitive Fragmentation Modeling for Metabolite Identification (CFM-ID)[3]. A peak in the predicted spectrum is considered to match a peak in the measured spectrum if the mass is within the specified mass tolerance (0.5 Da). The Jaccard score, which ranges between 0 and 1, is calculated as $|S_P \cap S_M| \div |S_P \cup S_M|$, where larger scores indicate a better peak match. The top five unknown metabolites, ranked by variable importance to PLS-DA projection scores, were annotated using CFM-ID, but fragmentation patterns between the unknown and candidate metabolites were not robust (Jaccard scores < 0.2). For comparison, panel a shows the best fit between measured and predicted spectra, and panel b shows the poorest fit. Jaccard scores and putative metabolite assignments are provided for each VIP-selected compound in Supplementary Table 3.

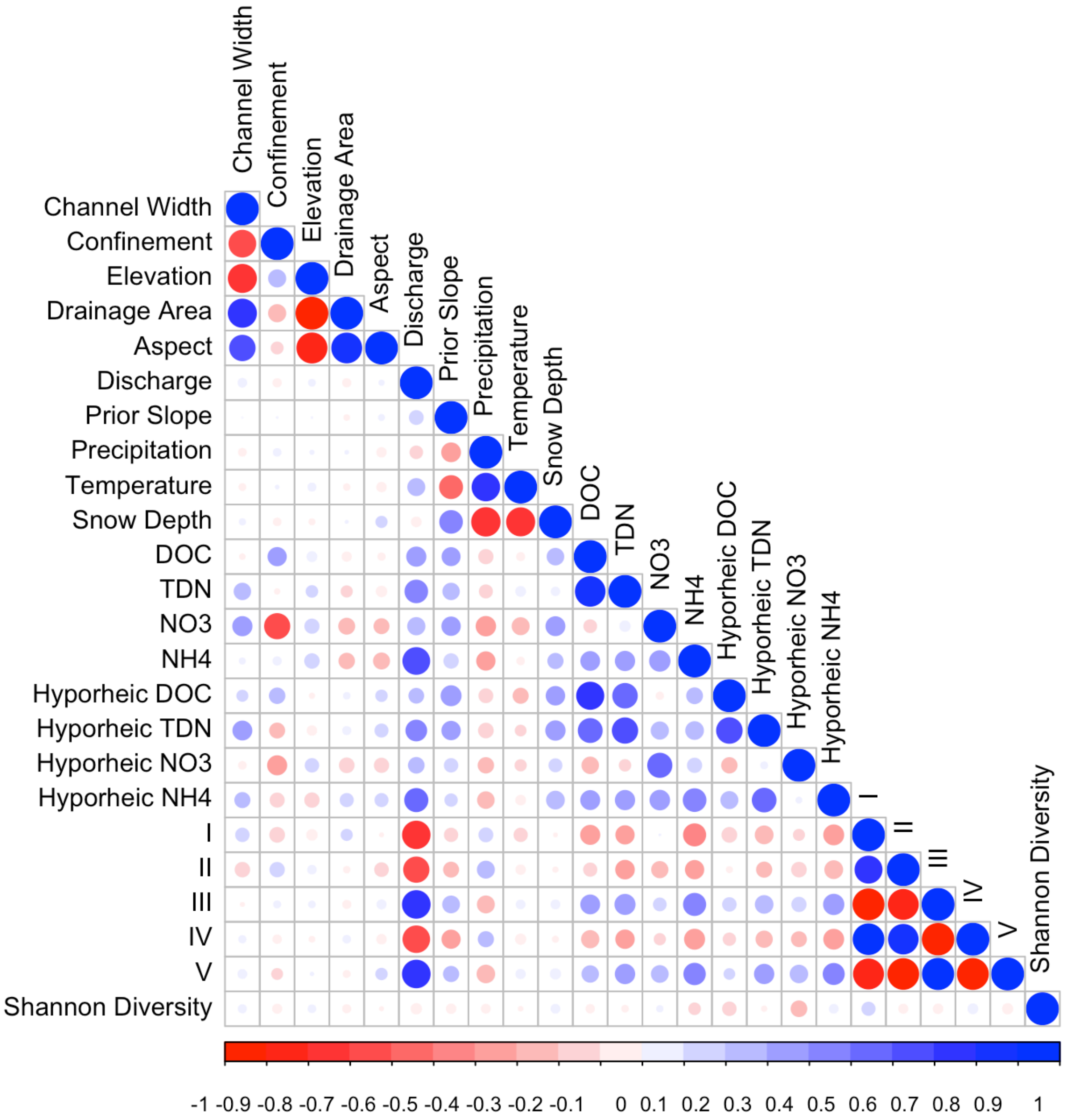

Supplementary Figure 10 | Correlation plot displays a matrix of potential predictor variables. The color scale indicates Spearman correlations between geomorphology, bulk chemistry, fluorescence spectroscopy, and the intensity of individual metabolites (red, negative; blue, positive). DOC, dissolved organic carbon; TDN, total dissolved nitrogen; NO3, nitrate; NH4, ammonium, Region I-V corresponds to EEMS indices; Shannon Diversity, calculated for normalized metabolites.

Supplementary Table 1 | Results of stepwise multiple linear regression models evaluating relationships between dissolved organic carbon (A), EEMS Region I (B), or EEMS Region III (C), with uncorrelated spatial and environmental components.

A) Dissolved organic carbon

	Estimate	Standard Error	t-value	p-value
(Intercept)	-3.57E+02	3.30E+02	-1.084	0.287
Width	-9.81E-03	9.85E-03	-0.996	0.326
Confinement	-1.42E-02	3.36E-03	-4.211	<0.001***
Aspect	8.05E-05	7.41E-05	1.086	0.285
Discharge	-2.05E-02	1.44E-02	-1.428	0.163
Precipitation	-5.72E-03	6.56E-03	-0.872	0.390
Temperature	7.25E-03	1.20E-02	0.603	0.551

Adjusted R^2: 0.29
F statistic: 3.54 on 6 and 32 DF
p-value: 0.01

B) EEMS Region I (simple aromatic proteins)

	Estimate	Standard Error	t-value	p-value
(Intercept)	-7.04E+03	8.69E+03	-0.810	0.424
Width	-1.02E-01	2.59E-01	-0.394	0.696
Confinement	-1.09E-01	8.85E-02	-1.235	0.226
Aspect	1.59E-03	1.95E-03	0.812	0.423
Discharge	-1.28E+00	3.78E-01	-3.372	0.002**
Precipitation	-1.46E-01	1.73E-01	-0.844	0.405
Temperature	-1.17E-02	3.17E-01	-0.037	0.971

Adjusted R^2: 0.27
F statistic: 3.36 on 6 and 32 DF
p-value: 0.01

C) EEMS Region III (fulvic-type acids)

	Estimate	Standard Error	t-value	p-value
(Intercept)	9.38E+03	1.49E+04	0.629	0.534
Width	1.07E-01	4.45E-01	0.239	0.812
Confinement	1.36E-01	1.52E-01	0.897	0.377
Aspect	-2.11E-03	3.35E-03	-0.628	0.534
Discharge	2.30E+00	6.49E-01	3.542	0.001**
Precipitation	-3.37E-02	2.97E-01	-0.114	0.910
Temperature	3.14E-01	5.44E-01	0.577	0.568

Adjusted R^2: 0.22
F statistic: 2.77 on 6 and 32 DF
p-value: 0.03

Supplementary Table 2 | Optical fluorescence characteristics of surface water and hyporheic sediments averaged across landscape position or collection period (discharge) at North St. Vrain (Pond, Upper, Meadow, Lower) and Upper Beaver Meadows (Simple). The average followed by the standard error (± 1 S.E.) in parentheses. EEMS regions I and II are related to simple aromatic proteins (tyrosine and tryptophan-like), region III to fulvic-acid type materials, region IV to soluble microbial byproduct-type materials, and region V to humic acid-type organics[8]. The level of significance from the 2-way ANOVA model including site (S) and discharge (D) are reported as * $p < 0.05$, ** $p < 0.01$, *** $p < 0.001$, or non-significant (ns). For ns comparisons, a master mean, integrated across landscape position or collection period, is provided. Specific comparisons were explored using Tukey HSD t-tests with Satterthwaite-Welsch approximations for degrees of freedom.

a) Landscape position

ID	I	II	III	IV	V
Simple					
Pond					
Upper	9.34 (1.29)	44.36 (0.93)	20.31 (2.21)	18.77 (0.84)	6.78 (0.75)
Meadow					
Lower					

b) Collection period

ID	I	II	III	IV	V
1	7.55 (1.56)	43.38 (1.76)	23.65 (3.17)	17.24 (0.97)	8.17 (1.12)
2	7.53 (0.66)	44.73 (0.52)	21.93 (1.27)	18.41 (0.56)	7.40 (0.37)
3	5.94 (0.26)	42.06 (0.31)	26.76 (0.04)	16.30 (0.03)	8.94 (0.15)
4	6.60 (0.59)	43.14 (0.55)	24.74 (0.98)	17.24 (0.32)	8.28 (0.37)
5	7.59 (0.60)	44.25 (0.66)	22.61 (1.29)	18.00 (0.62)	7.56 (0.47)
6	12.90 (1.61)	46.83 (1.87)	14.80 (3.69)	20.84 (1.56)	4.64 (1.14)
7	13.51 (0.96)	48.82 (1.17)	11.87 (2.26)	22.03 (0.83)	3.78 (0.66)
8	14.39 (1.87)	45.68 (0.70)	14.42 (2.07)	20.75 (0.93)	4.77 (0.69)

Source of variance

S	ns	ns	ns	ns	ns
D	***	**	***	***	***

Supplementary Table 3. Metabolites were ranked by variable importance to PLS-DA projection scores (VIP) for landscape position (panel a) and sampling period (panel b) using Metaboanalyst 3.0[2] (upper table). VIP scores are calculated as the weighted sum of squares of the PLS-DA loadings, accounting for the amount of Y-variation explained in each dimension. The ion intensity and *m/z* values are reported for the largest ion fragment within each clustered spectrum (where each spectrum represents a single TMS-derived metabolite). Lower *m/z* values indicate higher compound volatility and are typically associated with faster elution (retention times are reported in seconds). The F statistic and p-values are calculated using Tukey HSD posthoc t-tests. Putative metabolite identification and Jaccard scores were procured using Competitive Fragmentation Modeling for Metabolite Identification (CFM-ID), which provides a predicted ranking of possible candidate structures for a target spectrum[3]. However, fragmentation patterns between the unknown and candidate metabolites were not robust (Jaccard scores < 0.20). We identified putative parent compounds, molecular frameworks, and biological ontologies to provide class-level information for the unknown metabolites using Classyfire[4] and The Metabolomics Innovation Centre databases, but these frameworks should be interpreted with caution as the percent similarity between measured and predicted spectra was < 20% in all cases.

a) Landscape position

ID	*m/z*	Ion intensity	Retention time	F-value	Putative metabolite ID	Jaccard score	Putative parent class	Putative molecular framework	Putative biological ontology
115	514.38	1.98E+06	848.06	4.78**	4,4-Dimethyl-14alpha-formyl-5alpha-cholesta-8-en-3beta-ol	0.05	Triterpenoids	Aliphatic homopolycyclics	Lipid metabolism
249	415.24	6.55E+05	844.39	6.22***	(S)-Codamine	0.06	Benzylisoquinolines	Aromatic heteropolycyclics	Plant metabolite
193	289.17	1.96E+06	427.67	0.22	Methylguanidine	0.06	Guanidines	Aliphatic acyclics	Plant metabolite
147	219.14	1.53E+06	468.06	18.31***	1-Amino-propan-2-ol	0.17	Aminoalcohols	Aliphatic acyclics	Glycine, serine, threonine, and chlorophyll metabolism
62	322.18	1.98E+05	540.70	7.68***	Gemfibrozil	0.19	Phenol ethers	Aromatic homomonocyclics	Unknown environmental origin

b) Sampling period

ID	*m/z*	Ion intensity	Retention time	F-value	Putative metabolite ID	Jaccard score	Putative parent class	Putative molecular framework	Putative biological ontology
10	280.18	5.14E+06	837.35	4.25**	Eremopetasinorol	0.20	Cyclic alcohols and derivatives	Aliphatic homopolycyclics	Plant metabolite (cytoplasm & extracellular)
138	231.17	2.89E+06	838.55	11.68***	2-Pentyl-3-phenyl-2-propenal	0.11	Cinnamaldehydes	Aromatic homomonocyclics	Plant metabolite (cell membrane)
112	189.14	3.43E+07	915.86	32.63***	2-Diethylaminoethanol	0.11	Aminoalcohols	Aliphatic acyclics	Plant metabolite (cytoplasm & extracellular)
11	500.18	3.16E+05	754.91	10.55***	Lucernol	0.10	Coumestans	Aromatic heteropolycyclic compounds	Plant metabolite (flavonoid lipid)
33	460.28	4.79E+05	842.23	22.74***	Hydroxyprolyl-Leucine	0.17	Dipeptides	Aliphatic heteromonocyclics	Protein metabolism

Supplementary Table 3, continued.

a) Landscape position

ID	Putative metabolite ID	InChI identifier
115	4a-Formyl-5a-cholesta-8,24-dien-3b-ol	InChI=1S/C28H44O2/c1-18(2)7-6-8-19(3)22-11-12-23-20-9-10-24-21(17-29)26(30)14-16-28(24,5)25(20)13-15-27(22,23)4/h7,17,19,21-24,26,30H,6,8-16H2,1-5H3
249	4-Hydroxyatomoxetine	InChI=1S/C17H21NO2/c1-13-12-15(19)8-9-16(13)20-17(10-11-18-2)14-6-4-3-5-7-14/h3-9,12,17-19H,10-11H2,1-2H3/t17-/m1/s1
193	2,4-Diaminobutyric acid	InChI=1S/C4H10N2O2/c5-2-1-3(6)4(7)8/h3H,1-2,5-6H2,(H,7,8)/t3-/m0/s1
147	1-Methoxy-1H-indole-3-carboxaldehyde	InChI=1S/C10H9NO2/c1-13-11-6-8(7-12)9-4-2-3-5-10(9)11/h2-7H,1H3
62	Dimethylurea	InChI=1S/C3H8N2O/c1-4-3(6)5-2/h1-2H3,(H2,4,5,6)

b) Sampling period

ID	Putative metabolite ID	InChI identifier
10	2-Ethoxyethanol	InChI=1S/C4H10O2/c1-2-6-4-3-5/h5H,2-4H2,1H3
138	Isobutylpropylamine	InChI=1S/C7H17N/c1-4-5-8-6-7(2)3/h7-8H,4-6H2,1-3H3
112	1-Propylamine	InChI=1S/C3H9N/c1-2-3-4/h2-4H2,1H3
11	Phenytoin quinone	InChI=1S/C15H10N2O4/c18-11-7-6-10(8-12(11)19)15(9-4-2-1-3-5-9)13(20)16-14(21)17-15/h1-8H,(H2,16,17,20,21)
33	Glycyl-glycine	InChI=1S/C4H8N2O3/c5-1-3(7)6-2-4(8)9/h1-2,5H2,(H,6,7)(H,8,9)

Supplementary Table 4. Complete list of annotated metabolites collected from subalpine watersheds. Compounds were annotated by querying NIST Standard Reference and KEGG databases, and classified using ClassyFire, a taxonomic database. InChI identifiers were acquired using the PubChem database and are reported below.

Compound	InChi identifier
Lyxose	InChI=1S/C5H10O5/c6-2-1-10-5(9)4(8)3(2)7/h2-9H,1H2/t2-,3+,4+,5?/m1/s1
Gallic acid	InChI=1S/C7H6O5/c8-4-1-3(7(11)12)2-5(9)6(4)10/h1-2,8-10H,(H,11,12)
Dodecaethylene glycol	InChI=1S/C24H50O13/c25-1-3-27-5-7-29-9-11-31-13-15-33-17-19-35-21-23-37-24-22-36-20-18-34-16-14-32-12-10-30-8-6-28-4-2-26/h25-26H,1-24H2
2-hydroxypyridine	InChI=1S/C5H5NO/c7-5-3-1-2-4-6-5/h1-4H,(H,6,7)
Glycerol	InChI=1S/C3H8O3/c4-1-3(6)2-5/h3-6H,1-2H2
Triethylene glycol	InChI=1S/C6H14O4/c7-1-3-9-5-6-10-4-2-8/h7-8H,1-6H2
Galactose	InChI=1S/C6H12O6/c7-1-2-3(8)4(9)5(10)6(11)12-2/h2-11H,1H2/t2-,3+,4+,5-,6?/m1/s1
Phthalic acid	InChI=1S/C8H6O4/c9-7(10)5-3-1-2-4-6(5)8(11)12/h1-4H,(H,9,10)(H,11,12)
Tripropylene glycol mono-n-butyl ether	InChI=1S/C10H22O4/c1-2-3-5-12-7-9-14-10-8-13-6-4-11/h11H,2-10H2,1H3
Syringic acid	InChI=1S/C9H10O5/c1-13-6-3-5(9(11)12)4-7(14-2)8(6)10/h3-4,10H,1-2H3,(H,11,12)
Lumichrome	InChI=1S/C12H10N4O2/c1-5-3-7-8(4-6(5)2)14-10-9(13-7)11(17)16-12(18)15-10/h3-4H,1-2H3,(H2,14,15,16,17,18)
Palmitic acid	InChI=1S/C16H32O2/c1-2-3-4-5-6-7-8-9-10-11-12-13-14-15-16(17)18/h2-15H2,1H3,(H,17,18)
Arabinofuranose	InChI=1S/C5H10O5/c6-1-2-3(7)4(8)5(9)10-2/h2-9H,1H2/t2-,3-,4+,5+/m1/s1
Isovanillic acid	InChI=1S/C8H8O4/c1-12-7-3-2-5(8(10)11)4-6(7)9/h2-4,9H,1H3,(H,10,11)
Heptaethylene glycol	InChI=1S/C14H30O8/c15-1-3-17-5-7-19-9-11-21-13-14-22-12-10-20-8-6-18-4-2-16/h15-16H,1-14H2
Propylene glycol	InChI=1S/C3H8O2/c1-3(5)2-4/h3-5H,2H2,1H3
Benzoic acid	InChI=1S/C7H6O2/c8-7(9)6-4-2-1-3-5-6/h1-5H,(H,8,9)
Coumaric acid	InChI=1S/C9H8O3/c10-8-4-1-7(2-5-8)3-6-9(11)12/h1-6,10H,(H,11,12)/b6-3+
Sucrose	InChI=1S/C12H22O11/c13-1-4-6(16)8(18)9(19)11(21-4)23-12(3-15)10(20)7(17)5(2-14)22-12/h4-11,13-20H,1-3H2/t4-,5-,6-,7-,8+,9-,10+,11-,12+/m1/s1
Dodecanoic acid	InChI=1S/C10H20O2/c1-2-3-4-5-6-7-8-9-10(11)12/h2-9H2,1H3,(H,11,12)
Stearic acid	InChI=1S/C18H36O2/c1-2-3-4-5-6-7-8-9-10-11-12-13-14-15-16-17-18(19)20/h2-17H2,1H3,(H,19,20)
Glyceric acid	InChI=1S/C3H6O4/c4-1-2(5)3(6)7/h2,4-5H,1H2,(H,6,7)/t2-/m1/s1
Nonanoic acid	InChI=1S/C9H18O2/c1-2-3-4-5-6-7-8-9(10)11/h2-8H2,1H3,(H,10,11)
Silanol	InChI=1S/HOSi/c1-2/h1H
Thiazole	InChI=1S/C3H3NS/c1-2-5-3-4-1/h1-3H
Decaethylene glycol	InChI=1S/C20H42O11/c21-1-3-23-5-7-25-9-11-27-13-15-29-17-19-31-20-18-30-16-14-28-12-10-26-8-6-24-4-2-22/h21-22H,1-20H2

Supplementary references

1. Woolf, D., Lehmann, J. & Lee, D. R. Optimal bioenergy power generation for climate change mitigation with or without carbon sequestration. *Nat. Commun.* **7,** 1–11 (2016).
2. Xia, J. & Wishart, D. S. Using MetaboAnalyst 3.0 for Comprehensive Metabolomics Data Analysis. *Curr. Protoc. Bioinforma.* **55,** 10.1-14.10.91 (2016).
3. Allen, F., Pon, A., Wilson, M., Greiner, R. & Wishart, D. CFM-ID: A web server for annotation, spectrum prediction and metabolite identification from tandem mass spectra. *Nucleic Acids Res.* **42,** 94–99 (2014).
4. Feunang, Y. D. *et al.* ClassyFire: automated chemical classification with a comprehensive, computable taxonomy. *J. Cheminform.* **8,** 1–20 (2016).
